# KCNQ1 Potassium Channel Expressed in Human Sperm Is Involved in Sperm Motility, Acrosome Reaction, Protein Tyrosine Phosphorylation, and Ion Homeostasis During Capacitation

**DOI:** 10.3389/fphys.2021.761910

**Published:** 2021-10-22

**Authors:** Tian Gao, Kun Li, Fei Liang, Jianmin Yu, Ajuan Liu, Ya Ni, Peibei Sun

**Affiliations:** ^1^School of Pharmacy, Hangzhou Medical College, Hangzhou, China; ^2^Zhejiang Provincial Laboratory of Experimental Animal’s & Nonclinical Laboratory Studies, Hangzhou Medical College, Hangzhou, China

**Keywords:** KCNQ1 potassium channel, sperm capacitation, acrosome reaction, hyperactivation, ion homeostasis, protein tyrosine phosphorylation

## Abstract

Potassium channels are involved in membrane hyperpolarization and ion homeostasis regulation during human sperm capacitation. However, the types of potassium channels in human sperm remain controversial. The voltage-gated ion channel KCNQ1 is ubiquitously expressed and regulates key physiological processes in the human body. In the present study, we investigated whether KCNQ1 is expressed in human sperm and what role it might have in sperm function. The expression and localization of KCNQ1 in human sperm were evaluated using Western blotting and indirect immunofluorescence. During capacitation incubation, human sperm were treated with KCNQ1- specific inhibitor chromanol 293B. Sperm motility was analyzed using a computer-assisted sperm analyzer. The acrosome reaction was studied using fluorescein isothiocyanate-conjugated *Pisum sativum* agglutinin staining. Protein tyrosine phosphorylation levels and localization after capacitation were determined using Western blotting and immunofluorescence. Intracellular K^+^, Ca^2+^, Cl^−^, pH, and membrane potential were analyzed using fluorescent probes. The results demonstrate that KCNQ1 is expressed and localized in the head and tail regions of human sperm. KCNQ1 inhibition reduced sperm motility, acrosome reaction rates, and protein tyrosine phosphorylation but had no effect on hyperactivation. KCNQ1 inhibition also increased intracellular K^+^, membrane potential, and intracellular Cl^−^, while decreasing intracellular Ca^2+^ and pH. In conclusion, the KCNQ1 channel plays a crucial role during human sperm capacitation.

## Introduction

Freshly ejaculated human sperm cannot immediately fuse with an oocyte but must undergo a series of physiological and biochemical events known as capacitation, inside the female reproductive tract before they can fertilize an egg (De Jonge, 2017). This process is essential for natural fertilization. Sperm capacitation is accompanied by the removal of cholesterol from the membrane, membrane potential (V_m_) hyperpolarization, and intracellular alkalization, while increasing membrane permeability, intracellular calcium concentration ([Ca^2+^]_i_), and protein tyrosine phosphorylation levels (Battistone et al., 2013; Bernecic et al., 2019). After capacitation, sperm exhibit hyperactive motility and can undergo the acrosome reaction (AR). Hyperactive motility is a sperm swimming pattern with deep and asymmetrical flagellar bends, which helps sperm to progress toward and then penetrate an oocyte. The AR is a single-vesicle exocytotic event that facilitates sperm-oocyte fusion (Lopez-Torres and Chirinos, 2017). However, the mechanisms underlying capacitation remain unclear.

Ion channels play important roles in capacitation by regulating sperm membrane potential (V_m_), Ca^2+^ levels, and intracellular pH (pH_i_), which then affect AR, sperm motility, and other essential physiological processes involved in successful fertilization (Lishko et al., 2012; Brown et al., 2019). Potassium channels are crucial for sperm membrane potential hyperpolarization, ion homeostasis, and fertility (Vyklicka and Lishko, 2020). In mice, the principal K^+^ channel that mediates sperm membrane hyperpolarization during capacitation is Slo3, an intracellular alkalization-activated K^+^ channel, which is localized in the principal piece of the sperm tail region (Martinez-Lopez et al., 2009; Santi et al., 2010; Brenker et al., 2014). Mutations and deletions in *Slo3* affect male fertility (Zeng et al., 2011). In recent years, differences between human and mouse sperm K^+^ currents have been reported. The human sperm K^+^ current is activated by Ca^2+^ and is only weakly regulated by intracellular alkalization (Mannowetz et al., 2013). Mannowetz et al. proposed that Slo1 is the main K^+^ channel in human sperm, as the Slo1 channel is activated by Ca^2+^. They found that Slo1 is expressed and localized in the tail region of human sperm. K^+^ currents in human sperm can be inhibited by Slo1-specific inhibitors (Mannowetz et al., 2013). In addition, Brenker et al. suggested that Slo3 mediates human K^+^ currents. They found that as in case of Slo1, Slo3 is also expressed and localized in the tail region of human sperm and Slo3-specific inhibitors suppress human sperm K^+^ currents (Brenker et al., 2014). Lopez-Gonzalez et al. suggested that both Slo1 and Slo3 contribute to capacitation-mediated hyperpolarization based on pharmacological methods (Lopez-Gonzalez et al., 2014). However, Brown et al. reported an infertile patient whose sperm showed deficient K^+^ currents but still had intact *Slo1* and *Slo3* genes (Brown et al., 2016). Impaired assembly or localization of the Slo1/Slo3 channel could be an explanation, but the existence of other critical K^+^ channels in human sperm cannot be ruled out at this time. Taken together, current literature suggests that K^+^ channels in human sperm have not yet been fully elucidated.

KCNQ1 (also known as Kv7.1 or KvLQT1) is the pore-forming subunit (α subunit) of a voltage-gated K^+^ channel. It contains six transmembrane helices (S1-S6) and four intracellular C-terminal helices (HA-HD; Börjesson and Elinder, 2008; Catterall, 2010; Sun and MacKinnon, 2017). The S1-S4 segments constitute the voltage-sensing domain (VSD), which controls the opening of the channel. The S5-S6 segments constitute the pore domain (PD), which has K^+^ selectivity (Dixit et al., 2020) KCNQ1 forms a tetramer and performs its important physiological functions by interacting with auxiliary subunits, including KCNE family members (KCNE1-5; Nakajo and Kubo, 2015; Sun and MacKinnon, 2020). KCNQ1 is expressed in a wide range of human tissues, including the heart, kidney, colon, cochlea, stomach, and small intestine (Bett et al., 2006; Dixit et al., 2020). In different tissues, KCNQ1 interacts with different auxiliary subunits and performs different functions. For example, in the human heart, KCNQ1 interacts with KCNE1 and mediates a delayed rectifier K^+^ current, which is critical for cardiac action potential repolarization (Wu and Larsson, 2020). In the human stomach, KCNQ1 forms a functional channel with KCNE2 and regulates gastric acid secretion (Roepke et al., 2006). Previous studies have shown that KCNQ1 and KCNE1 are expressed in rat testes and germ cells (Tsevi et al., 2005). The auxiliary subunit KCNE1 is also expressed and localized in the tail region of human sperm (Yeung and Cooper, 2008), suggesting that its pore-forming subunit KCNQ1 may also be expressed in human sperm. KCNQ1 C-terminal intracellular helices, HA and HB, can interact with calmodulin (CAM), a cytosolic Ca^2+^-binding protein that affects KCNQ1 function. Reduced [Ca^2+^]_i_ causes inactivation of the KCNQ1 channel (Sun and MacKinnon, 2017). Therefore, we hypothesized that KCNQ1 exists in human sperm and plays a role in sperm function. The findings of this research will help in further understanding the role of K^+^ channels in human sperm capacitation.

## Materials and Methods

### Chemicals and Reagents

Percoll was obtained from GE Healthcare BioSciences (Little Chalfont, UK). Lysis buffer and other reagents for sodium dodecyl sulfate-polyacrylamide gel electrophoresis (SDS-PAGE) were purchased from the Beyotime Institute of Biotechnology (Shanghai, China). Dimethyl sulfoxide (DMSO) was acquired from Merck (Darmstadt, Germany). Enhanced chemiluminescence (ECL) Plus Chemiluminescence Kit, protein loading buffer, and pre-dyed protein markers were acquired from Thermo Fisher Scientific (Burlington, NC, United States). Polyvinylidene fluoride (PVDF) membranes were obtained from Millipore Corporation (Bedford, MA, United States). Fluorescein isothiocyanate-conjugated *Pisum sativum* agglutinin (PSA-FITC), KCNQ1 inhibitor chromanol 293B, and carbonyl cyanide m-chlorophenylhydrazone (CCCP) were obtained from Sigma-Aldrich (St. Louis, MO, United States). Complete mini EDTA-free protease inhibitor cocktail and phosphatase inhibitor cocktail (broad-spectrum phosphatase inhibitor, including Ser/Thr and Tyr phosphatase inhibitors) were obtained from Roche (Mannheim, Germany). The antibodies used in this study were as follows: KCNQ1 (ab84819), KCNE1 (ab65795), rabbit anti-β-tubulin (ab6046), Alexa Fluor 555-conjugated goat anti-mouse antibody (ab150118), Alexa Fluor 488-conjugated goat anti-rabbit antibody (ab150077), and Alexa Fluor 488-conjugated goat anti-mouse antibody (ab150113) were purchased from Abcam (Cambridge, UK); p-Tyr (sc-7,020) and KCNQ1 (sc-365,764) were obtained from Santa Cruz Biotechnology Inc (Dallas, Texas, United States). KCNE1 (31195A31) and horseradish peroxidase-conjugated goat antibodies were purchased from Invitrogen (Carlsbad, CA, United States). Fluo3-AM and MQAE were obtained from Beyotime Institute of Biotechnology (Shanghai, China). BCECF-AM, DisC3(5), and PBFI-AM were purchased from Invitrogen (Carlsbad, CA, United States).

### Sperm Incubation Medium

Human tubal fluid (HTF) medium was prepared as previously described (Li et al., 2021; Sun et al., 2021). The HTF medium comprised 5.06mM KCl, 90mM NaCl, 25.3mM NaHCO_3_, 1.17mM KH_2_PO_4_, 1.8mM CaCl_2_, 1.01mM MgSO_4_, 0.27mM sodium pyruvate, 5.56mM glucose, 21.6mM sodium lactate, 20mM HEPES, 4g/L bovine serum album, 5mg/L phenol red and 60mg/L penicillin. The pH was adjusted to 7.4. All chemicals were obtained from Sigma-Aldrich.

### Semen Collection and Sample Preparation

This study was approved by the Medical Ethics Committee of Hangzhou Medical College (no. 2018004). Written informed consent was obtained from 15 healthy male donors (aged 25–35years). Sperm preparation was performed as previously described (Sun et al., 2021). The donors abstained from sexual intercourse for 3days before sample collection. Fresh semen were obtained *via* masturbation, collected in sterile containers, and subsequently liquefied at 37°C for 1h. According to the World Health Organization (WHO) requirements, semen samples in this study met the following criteria: sperm viability ≥85%, sperm motility ≥50%, morphologically normal sperm ≥15%, and sperm concentration≥20×10^6^ sperm/mL. To remove dead sperm and cell debris, semen samples were centrifuged with 40 and 80% discontinuous Percoll gradients at 750×*g* for 15min and the precipitate was resuspended in HTF medium. Sperm collected from at least three donors were mixed, washed, adjusted to a density of approximately 20×10^6^ sperm/mL, and analyzed in the following experiments. The prepared samples were incubated in a 5% CO_2_ incubator at 37°C.

### Protein Extraction and Western Blotting

According to our previously reported method (Sun et al., 2021), sperm samples were washed with phosphate-buffered saline (PBS) and resuspended in lysis buffer (P0013G, Beyotime Institute of Biotechnology, Shanghai, China) containing protease inhibitors (protease inhibitor cocktail and phosphatase inhibitor cocktail, Roche, Mannheim, Germany) and 1mM phenylmethylsulfonyl fluoride (PMSF). After ultrasonication and centrifugation, the supernatant was collected. Protein sample concentrations were determined using a bicinchoninic acid assay (BCA) kit (Beyotime Institute of Biotechnology, Shanghai, China). For different treatment groups, equal amounts of sperm protein (20μg) were denatured *via* incubation with protein loading buffer at 100°C for 5min and separated by 10% SDS-PAGE with a pre-stained protein marker. Proteins, transferred to PVDF membranes, were blocked with 5% skim milk (m/v). The PVDF membranes were incubated with primary antibodies at 4°C overnight and then washed three times with TBS buffer supplemented with 0.01% Tween-20 (v/v). The membrane was incubated with appropriate secondary antibodies at room temperature for 2h. After washing with TBS buffer three times, protein blots were detected by an ECL kit (Thermo Fisher Scientific) using a gel imaging system (Amersham Imager 600; General Electric Company, United States). For loading control, the membranes were stripped and probed with β-tubulin antibodies. Gray intensity was analyzed using the ImageJ software.

### Indirect Immunofluorescence Staining

After fixation in 4% paraformaldehyde for 30min, the sperm were mounted on Silane-Prep slides and airdried. Sperm were permeabilized with 0.1% Triton X-100 and blocked with 10% goat serum. The sperm were then incubated with primary antibodies (mouse anti-KCNQ1, rabbit anti-KCNE1 or mouse anti-p-Tyr) or normal IgG (negative control) overnight at 4°C. After washing three times with PBS, Alexa Fluor 555-conjugated anti-mouse IgG secondary antibody and Alexa Fluor 488-conjugated anti-rabbit IgG secondary antibody were applied for 1h at 37°C. Following incubation with DAPI and washing with PBS, the sperm were examined using fluorescence microscopy (Nikon Eclipse 80i; Nikon Inc., Tokyo, Japan). For the immunofluorescence studies of KCNQ1, KCNE1 and p-Tyr, both non-capacitation and capacitation for 3h samples were used.

### Evaluation of Sperm Capacitation and Sperm Viability

Because only capacitated sperm undergo exocytosis, human sperm capacitation was assessed indirectly using progesterone-induced AR. Different sperm groups were treated with different reagents for 3h during capacitation, followed by treatment with 15μM progesterone for 15min to induce the AR. According to the WHO Laboratory Manual for the Examination and Processing of Human Semen (5th ed.), the AR was evaluated by PSA-FITC staining. After fixing with 95% ethanol for 30min, sperm were mounted on Silane-Prep slides, air dried, and incubated overnight at 4°C with 25mg/L PSA-FITC in the dark. Sperm were washed with PBS and analyzed by fluorescence microscopy. At least 200 sperm were counted for each sample. To detect spontaneous AR, sperm were stained with PSA-FITC immediately after discontinuous Percoll gradient centrifugation and washing.

To evaluate sperm viability, propidium iodide (PI) was used to detect dead cells. Sperm were stained with 12μM PI for 10min at 37°C before or after capacitation for 3h. After washing with PBS three times, the sperm were mounted on Silane-Prep slides, air dried, and analyzed by fluorescence microscopy. Sperm with red fluorescence at the head was considered dead sperm. At least 200 sperm were counted for each sample. The percentage of non-viable cells (NVC%) was calculated.

### Sperm Motility Analysis

Sperm motility was analyzed using a computer-assisted sperm analyzer (CASA; IVOS, Hamilton-Thorne Bio-Sciences, Beverly, MA, United States) with the following parameters: acquisition frame, 30; frame rate, 60Hz; minimum cell size, 3 pixels; minimum contrast, 80; cell intensity, 40; magnification, 1.73 ×; temperature, 37°C; illumination intensity, 2,164; path velocity, 25.0μm/s; straightness threshold, 80%; slow cell, average path velocity (VAP) and straight line velocity (VSL) of less than 5.0μm/s and 11μm/s, respectively; and chamber depth, 20μm (*n*>200 motile sperm per sample). Briefly, a 5μL sperm sample was loaded into a 20μm deep slide chamber warmed to 37°C. The following parameters were assessed for each sample: VSL, VAP, curvilinear velocity (VCL), straightness (STR), linearity (LIN), amplitude of lateral head displacement (ALH), beat-cross frequency (BCF), and percentage of motile, progressive, and hyperactivation. Hyperactivated sperm met the following criteria: VCL≥150μm/s, ALH≥7.0μm, and LIN≤50%.

### Intracellular K^+^ Measurement in Human Sperm

[K^+^]_i_ in sperm was measured using PBFI-AM. Sperm were loaded with 10μM PBFI-AM in a 5% CO_2_ incubator in the dark at 37°C for 30min. Excess dye in the medium was removed by washing five times with HTF. PBFI-AM-loaded sperm were resuspended in HTF and incubated at 37°C for further 20min. Sperm aliquots (10^6^ cells/mL) were exposed to vehicle control (DMSO) and chromanol 293B (20, 100, or 200μM). The K^+^ fluorescence signal was then recorded using a Synergy 2 Multi-Function Microplate Reader (Bio-Tek Instruments, Winooski, United States) with excitation at 340/380nm and emission wavelengths of 500nm. The data were acquired at 3min intervals for 30min during capacitating incubation because the effect of chromanol 293B on ion homeostasis may be compensated for by other potassium channels over time. After capacitation for 3h, a fluorescence signal was acquired. The ratio (340:380) of the two signals is directly proportional to [K^+^]_i_. First recorded raw intensity values were used to normalize the other raw intensity values.

### Intracellular Ca^2+^ Measurement in Human Sperm

The [Ca^2+^]_i_ levels in human sperm were measured using Fluo3-AM according to a previously described method (Li et al., 2014). Briefly, the prepared sperm were loaded with 10μM Fluo3-AM in a 5% CO_2_ incubator at 37°C for 30min in the dark and then washed five times with HTF to remove free Fluo3-AM. Fluo3-AM-loaded sperm were resuspended in HTF and incubated at 37°C for another 20min. Sperm aliquots (10^6^ cells/mL) were then exposed to vehicle control (DMSO) and chromanol 293B (20, 100, or 200μM). The Ca^2+^ fluorescence signal was then recorded using a Synergy 2 Multi-Function Microplate Reader, at 485nm excitation and 528nm emission wavelengths. The data were acquired at 3min intervals for 30min during the capacitating incubation. After capacitation for 3h, a fluorescence signal was acquired. Fluorescence intensity is directly proportional to [Ca^2+^]_i_. First recorded raw intensity values were used to normalize the other raw intensity values.

### Intracellular Cl^−^ Measurement in Human Sperm

The [Cl^−^]_i_ levels in sperm were measured using a Cl^−^- specific fluorescence probe (MQAE). Prepared sperm were loaded with 5μM MQAE in a 5% CO_2_ incubator in the dark at 37°C for 30min and were then washed five times with HTF to remove free MQAE. MQAE-loaded sperm were resuspended in HTF and incubated at 37°C for another 20min. MQAE-loaded sperm aliquots (10^6^ cells/mL) were exposed to vehicle control (DMSO) and chromanol 293B (20, 100, or 200μM). The Cl^−^ fluorescence signal was recorded using a Synergy 2 Multi-Function Microplate Reader at 355nm excitation and 460nm emission wavelengths. The data were acquired at 3min intervals for 30min during the capacitating incubation. After capacitation for 3h, a fluorescence signal was acquired. Fluorescence intensity was inversely proportional to [Cl^−^]_i_. First recorded raw intensity values were used to normalize the other raw intensity values.

### Intracellular pH Measurement in Human Sperm

Sperm sample pH was evaluated using BCECF-AM. The prepared sperm were loaded with 10μM BCECF-AM in a 5% CO_2_ incubator in the dark at 37°C for 30min and were then washed five times with HTF to remove free BCECF-AM. BCECF-AM-loaded sperm were resuspended in HTF and incubated at 37°C for another 20min. BCECF-AM-loaded sperm aliquots (10^6^ cells/mL) were exposed to vehicle control (DMSO) and chromanol 293B (20, 100, or 200μM). To determine pH_i_, fluorescence signals were recorded using a Synergy 2 Multi-Function Microplate Reader at 490/440nm excitation and 535nm emission wavelengths. The data were acquired at 3min intervals for 30min during the capacitating incubation. After capacitation for 3h, a fluorescence signal was acquired. The ratio (490:440) of the two signals was directly proportional to the pH_i_. The first recorded raw intensity values were used to normalize the other raw intensity values.

### Assessment of Sperm Membrane Potential Changes

Sperm membrane potential changes were evaluated using the potential-sensitive fluorescence probe DiSC3(5), as previously described (Xu et al., 2007). Before measurement, the prepared sperm were loaded with 1μM DisC3(5) in a 5% CO_2_ incubator in the dark at 37°C for 5min. CCCP was added to a final concentration of 1μM. Sperm were incubated for 2min. Sperm aliquots (10^6^ cells/mL) were exposed to vehicle control (DMSO) and chromanol 293B (20, 100, or 200μM). The membrane potential fluorescence signal was then recorded using a Synergy 2 Multi-Function Microplate Reader, with 620nm excitation and 670nm emission wavelengths. The data were acquired at 3min intervals for 30min during the capacitating incubation. After capacitation for 3h, a fluorescence signal was acquired. The fluorescence intensity was directly proportional to V_m_. The first recorded raw intensity values were used to normalize the other raw intensity values.

### Statistical Analysis

The Statistical Package for the Social Sciences software (SPSS, version 23; IBM Corporation, Armonk, NY, United States) was used for statistical analyses. Results are expressed as the means ± standard error of the mean (SEM). One-way analysis of variance was used to determine differences between the groups. When tests for the homogeneity of variance were not significant, the least significant difference test was used; otherwise, the data were analyzed using Dunnett’s T3 test; and *p*<0.05 was considered statistically significant (two-sided).

## Results

### Expression and Localization of KCNQ1 and KCNE1 in Human Sperm

**Figure 1:**
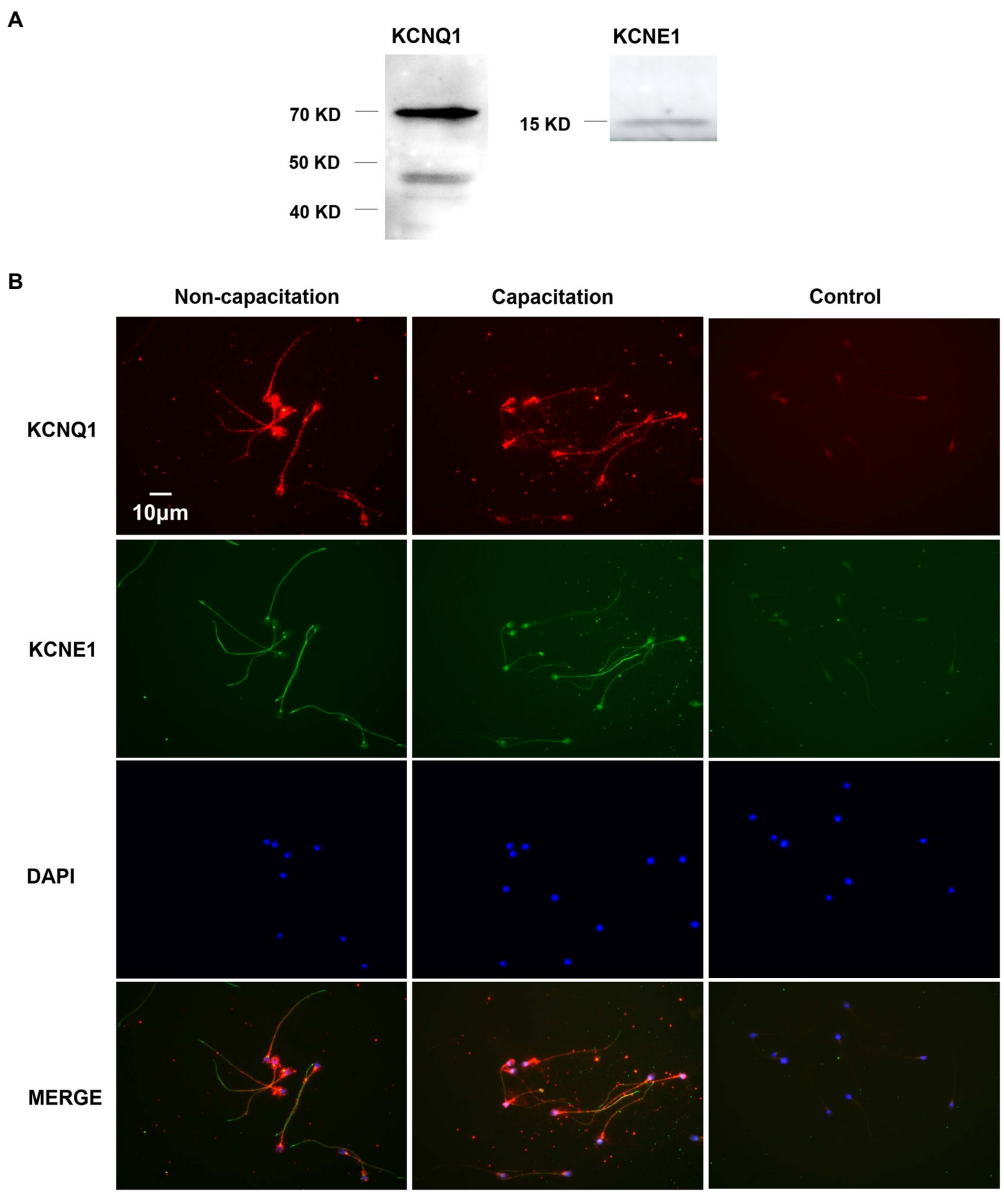
Expression and localization of KCNQ1 and KCNE1 in human sperm. **(A)** Human sperm were lysed after capacitation for 3h. Sperm proteins were separated and analyzed using 10% SDS-PAGE and Western blotting, using antibodies for KCNQ1 (Abcam) and KCNE1 (Abcam). The figure is representative of 3 separate experiments. The full uncropped immunoblots was provided in the supplementary data (Supplementary Figure 1). **(B)** Indirect immunofluorescence of KCNQ1 (Santa Cruz) and KCNE1 (Invitrogen) in human sperm before and after capacitation. KCNQ1 (red), KCNE1 (green), and co-localization of KCNQ1 and KCNE1 (merge). The negative control cells were incubated with normal IgG as the primary antibodies. The nuclei of spermatozoa were stained blue with DAPI. The figure is representative of 3 separate experiments.

We studied the expression of KCNQ1 and KCNE1 in human sperm using Western blotting. The results showed the presence of a KCNQ1-specific band at approximately 70kDa and nonspecific band at approximately 48kDa, in addition to a KCNE1-specific band at 15kDa (Figure 1A). We examined the immunofluorescence of KCNQ1 and KCNE1 in human sperm before and after capacitation (Figure 1B). The results showed that KCNQ1 was localized mainly in the head and tail regions of human sperm, while KCNE1 was localized mainly in the neck and tail regions, which is in accordance with previous studies (Yeung and Cooper, 2008). The merging of both proteins showed that they were partially co-localized. The localization of KCNQ1 and KCNE1 in human sperm before and after capacitation was not significantly different. These results show that both KCNQ1 and KCNE1 are expressed in human sperm.

### Chromanol 293B Affects AR During Human Sperm Capacitation

**Figure 2:**
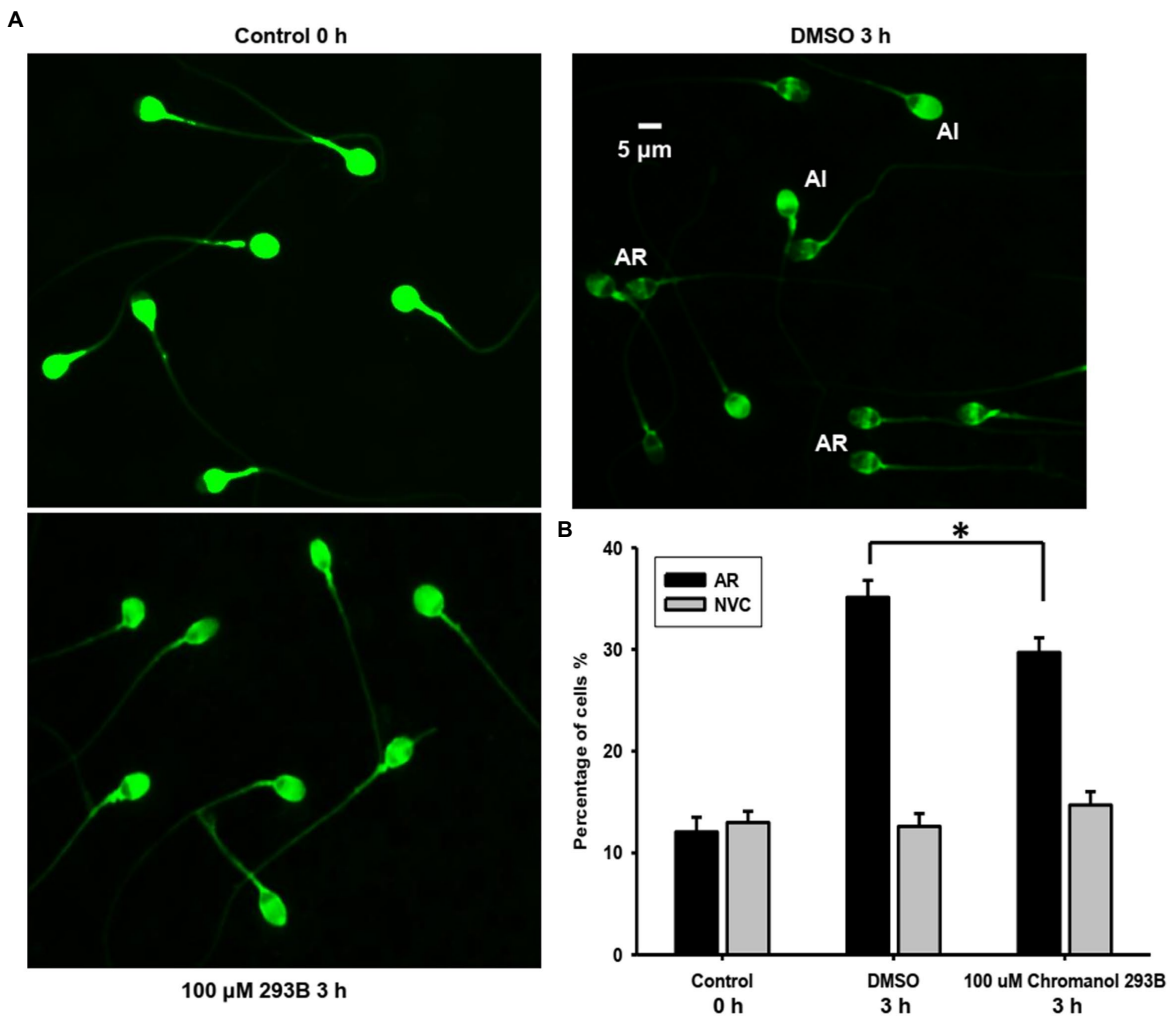
Chromanol 293B effect on human sperm AR and viability. **(A)** The human sperm AR was evaluated using PSA-FITC staining. Sperm were treated with different reagents (DMSO or 100μM chromanol 293B) for 3h during capacitation, and the sperm were then treated with 15μM progesterone for 15min to induce AR. To detect spontaneous AR before capacitation, sperm were stained with PSA-FITC immediately after discontinuous Percoll gradient centrifugation and washing. AR indicates acrosome-reacted sperm, while acrosome integrity (AI) indicates uncapacitated sperm. **(B)** After PSA-FITC staining, sperm were examined by fluorescence microscopy. The AR ratio is calculated by dividing the number of AR sperm with the total number of sperm. For 100 μM chromanol 293B group compared to vehicle control group, ^*^p<0.05. Propidium iodide (PI) is used to stain dead sperm. Percentage of not viable cells (NVC%) was calculated. Values represent the means ± SEM of at least 6 experiments.

The effect of chromanol 293B on the human sperm AR was detected using a PSA-FITC staining assay. This method can easily distinguish sperm with acrosome integrity (AI) from those with AR (Figure 2A). For the AI pattern, bright and uniform fluorescence was observed in most regions of sperm heads, whereas for the AR pattern, no fluorescent staining was observed in the acrosomal zone, or only fluorescence bands in the equatorial zone were observed. The spontaneous AR (control 0h) ratio was approximately 12.1±1.4%, which was determined before capacitation incubation. After capacitation for 3h, the AR ratio of vehicle control (DMSO) was approximately 35.1±1.7%. When human sperm were treated with 100μM chromanol 293B during capacitation, the ratio of AR was significantly lower than that of the vehicle control (29.7±1.4% vs. 35.1±1.7%, *p*=0.017; Figure 2B). However, chromanol 293B did not affect the sperm viability. As shown in Figure 2B, before capacitation, the number of NVC% was approximately 13.0±1.1%. After capacitation for 3h, there were no significant differences in NVC% between the vehicle control group and the 100μM chromanol 293B-treated group (12.6±1.3% vs. 14.7±1.3%). These results suggest that the KCNQ1 channel is involved in human sperm capacitation.

### Chromanol 293B Changes Human Sperm Motility Parameters During Capacitation

CASA was used to analyze the effects of chromanol 293B on human sperm motility. As shown in Table 1, no significant differences were observed in human sperm hyperactivation between the chromanol 293B-treated groups and vehicle control (DMSO) for 3h capacitation. However, most sperm motility parameters in the 200μM chromanol 293B group were significantly lower than those in the vehicle control group, including sperm motility, progressive sperm, VAP, VSL, VCL, BCF, STR, and LIN (*p*<0.05). When sperm were treated with 100μM chromanol 293B, only sperm motility and progressive sperm showed significant differences compared to the vehicle control. These results suggest that KCNQ1 plays a role in human sperm motility during capacitation.

**Table 1:** Effect of chromanol 293B on motility parameters during human sperm capacitation. Values represent the means ± SEM of 5 experiments.

	DMSO	100μM Chromanol 293B	200μM Chromanol 293B
Motility (%)	67.06 ± 1.33	63.26 ± 0.80^*^	34.30 ± 1.24^*^
Progressive motility (%)	46.76 ± 1.13	42.89 ± 0.89^*^	21.90 ± 0.66^*^
VAP (μm/s)	81.81 ± 1.64	78.52 ± 1.40	70.17 ± 1.07^*^
VSL (μm/s)	72.25 ± 1.62	69.01 ± 1.33	59.46 ± 0.95^*^
VCL (μm/s)	130.33 ± 2.66	126.99 ± 2.78	118.95 ± 1.91^*^
ALH (μm)	5.65 ± 0.14	5.52 ± 0.14	5.91 ± 0.07
BCF (Hz)	31.48 ± 0.29	32.01 ± 0.31	25.67 ± 0.29^*^
STR (%)	85.12 ± 0.42	84.50 ± 0.38	81.90 ± 0.31^*^
LIN (%)	54.06 ± 0.44	53.10 ± 0.38	49.30 ± 0.45^*^
Hyperactivation(%)	7.00 ± 0.54	7.01 ± 0.82	7.70 ± 0.68

* *p<0.05*.

*VAP, average path velocity; VSL, straight line velocity; VCL, curvilinear velocity; ALH, amplitude of lateral head displacement; BCF, beat-cross frequency; STR, straightness (VSL/VAP multiplied by 100); LIN, linearity (VSL/VCL multiplied by 100); Hyperactivation, VCL≥150μm/s, ALH≥7.0μm, and LIN≤50%*.

### Chromanol 293B Changes Protein Tyrosine Phosphorylation Level and Localization During Human Sperm Capacitation

**Figure 3:**
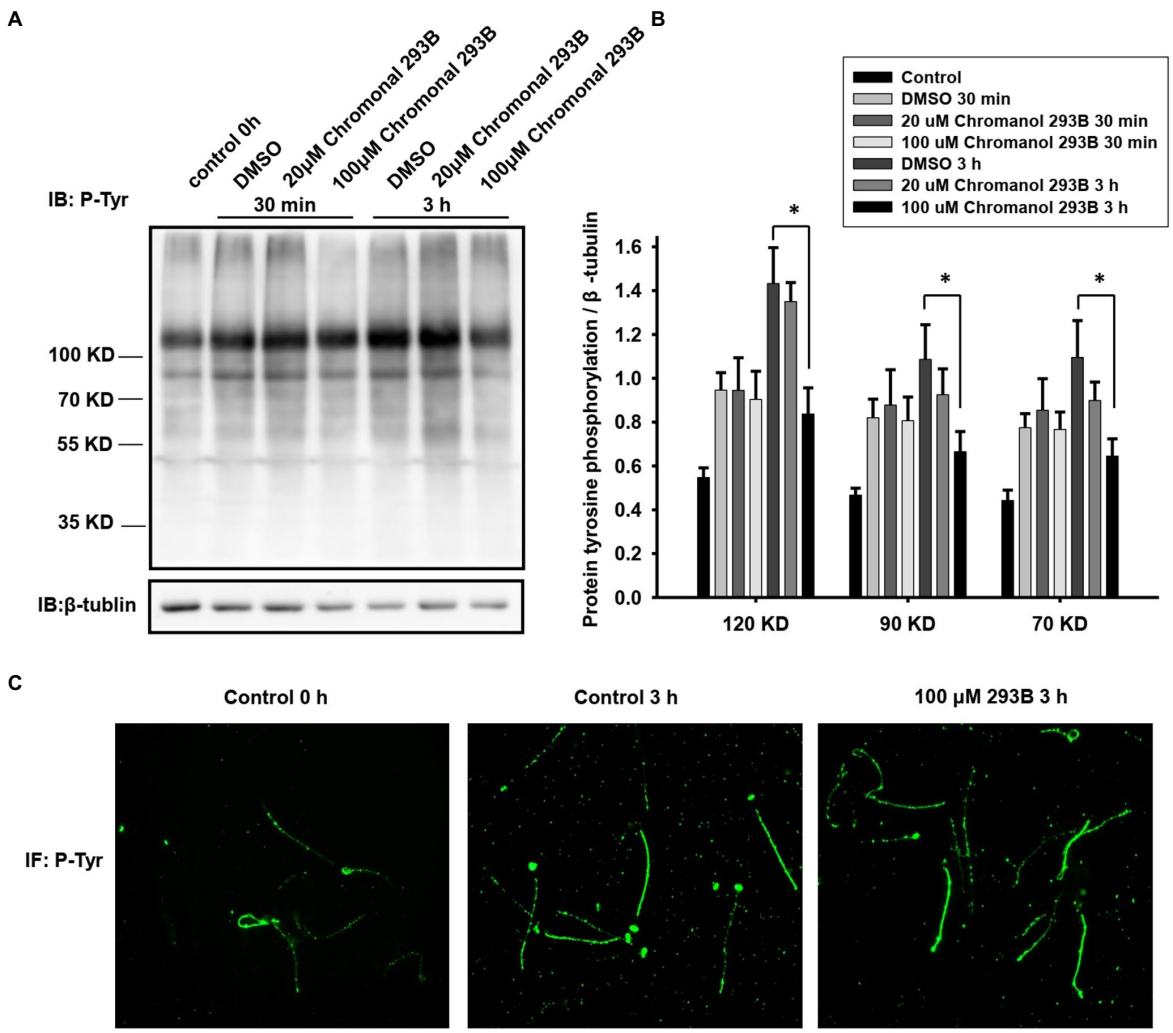
Effect of chromanol 293B on protein tyrosine phosphorylation during human sperm capacitation. **(A)** Western blotting results showing the effect of chromanol 293B on protein tyrosine phosphorylation during human sperm capacitation. Sperm before capacitation incubation were used as the control group. Other sperm were treated with the vehicle control (DMSO), 20μM chromanol 293B, and 100μM chromanol 293B under capacitated conditions for 30min and 3h. Thereafter, sperm were lysed and then the proteins were resolved using 10% SDS-PAGE. Protein tyrosine phosphorylation was detected using a primary anti-phosphotyrosine antibody by Western blotting. Subsequently, the blot was stripped and probed with an anti-β-tubulin antibody as a loading control. The figure is representative of 6 separate experiments. The full uncropped immunoblots was provided in the supplementary data (Supplementary Figure 2). **(B)** The ratio of protein tyrosine phosphorylation levels to that of the loading control, β-tubulin. Values represent the means ± SEM of 6 experiments. For 100μM chromanol 293B group compared to vehicle control group, ^*^p<0.05. **(C)** Indirect immunofluorescence of protein tyrosine phosphorylation in human sperm before and after capacitation for 3h. The figure is representative of 3 separate experiments.

To further verify that the KCNQ1 potassium channel plays a role in human sperm during capacitation, we examined the effect of chromanol 293B on protein tyrosine phosphorylation levels, because capacitated sperm show high levels of protein tyrosine phosphorylation (Visconti et al., 2011). The effect of chromanol 293B on protein tyrosine phosphorylation levels was assessed by Western blotting. As shown in Figure 3A, protein tyrosine phosphorylation levels increased during human sperm capacitation. After 3h of sperm capacitation, the 100μM chromanol 293B group showed a significant decrease in protein tyrosine phosphorylation. We analyzed the ratio of protein tyrosine phosphorylation levels to that of the loading control, β-tubulin (Figure 3B). Protein tyrosine phosphorylation was enhanced over time during capacitation. The effect of 30min treatment of chromanol 293B on capacitation was negligible. However, after capacitation for 3h, chromanol 293B reduced protein tyrosine phosphorylation levels in a dose-dependent manner. The addition of 100μM chromanol 293B significantly decreased tyrosine phosphorylation, with bands at 120, 90, and 70kDa compared to the DMSO control (*p*=0.005, 0.012, and 0.032, respectively). We also examined the effect of chromanol 293B on phosphorylated tyrosine localization using indirect immunofluorescence. Before capacitation, phosphorylated tyrosine was localized in the head and tail regions of the sperm, and the fluorescence intensity was weak. After capacitation for 3h, the phosphorylated tyrosine was mainly localized in the equatorial and tail regions of the sperm, and the fluorescence intensity became intense. However, when the sperm were treated with 100μM chromanol 293B, phosphorylated tyrosine only localized in the tail region (Figure 3C).

### Chromanol 293B Increases Intracellular K^+^ Concentration During Human Sperm Capacitation

**Figure 4:**
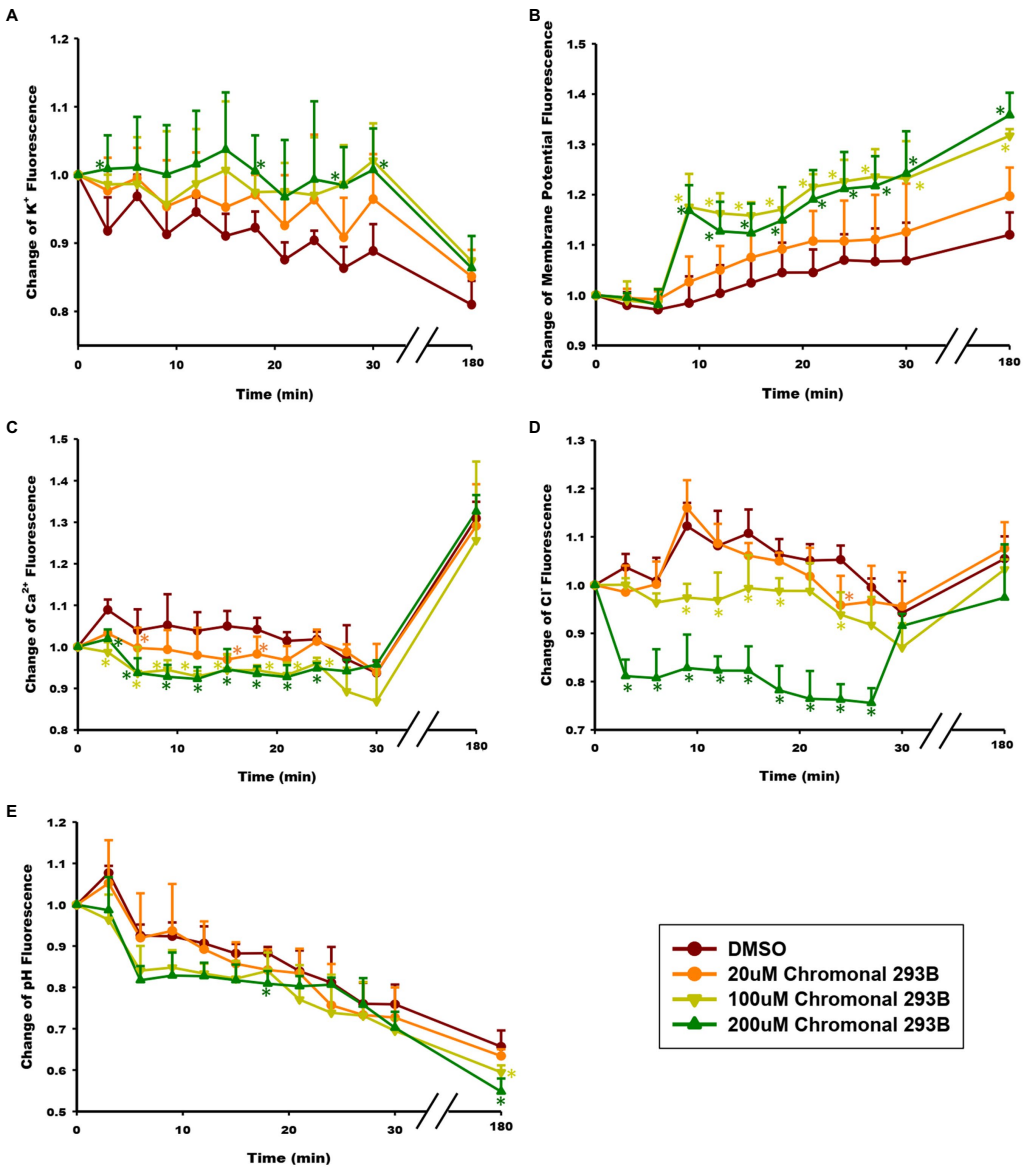
Effect of chromanol 293B on ion homeostasis during human sperm capacitation. **(A)** Intracellular K^+^ concentration ([K^+^]_i_) was detected using the K^+^ fluorescence probe PBFI-AM. The fluorescence intensity was directly proportional to [K^+^]_i_. **(B)** Human sperm membrane potential (V_m_) was analyzed using the fluorescence probe DisC3(5). The fluorescence intensity was directly proportional to V_m_. **(C)** Intracellular Ca^2+^ concentration ([Ca^2+^]_i_) was detected using the Ca^2+^ fluorescence probe Fluo3-AM. The fluorescence intensity was directly proportional to [Ca^2+^]_i_. **(D)** Intracellular Cl^−^ concentration ([Cl^−^]_i_) was detected using the Cl^−^ fluorescence probe MQAE. The fluorescence intensity was inversely proportional to [Cl^−^]_i_. **(E)** Intracellular pH (pH_i_) was analyzed using the pH fluorescence probe BCECF-AM. The fluorescence intensity was directly proportional to pH_i_. All the data were acquired at 3min intervals for 30min during capacitating incubation. After capacitation for 3h, the fluorescence signal was acquired again. Asterisks indicate the significant differences between samples and vehicle control at the same time point (^*^p<0.05). The exact P value were provided in the supplementary data (Table S1). Values represent the means ± SEM of 3 experiments.

To determine whether the KCNQ1 channel mediates K^+^ currents during human sperm capacitation, the effect of chromanol 293B on intracellular K^+^ concentration ([K^+^]_i_) was examined. [K^+^]_i_ was analyzed at 3min intervals over 30min and once after sperm were capacitated for 3h. The intracellular K^+^ concentration was detected using the K^+^ fluorescence probe PBFI-AM, and the fluorescence intensity was directly proportional to [K^+^]_i_. As shown in Figure 4A, [K^+^]_i_ decreased during human sperm capacitation in the vehicle control (DMSO), suggesting that potassium channels open and result in K^+^ efflux. However, [K^+^]_i_ increased when sperm were treated with chromanol 293B. The results showed dose-dependent changes, suggesting that chromanol 293B inhibits the KCNQ1 potassium channel, blocks K^+^ efflux through KCNQ1, and increases [K^+^]_i_.

### Chromanol 293B Depolarizes Membrane Potential During Human Sperm Capacitation

Because KCNQ1 inhibition increases [K^+^]_i_ in sperm and potassium channels are always involved in the regulation of membrane potential (V_m_), we further examined whether the membrane was depolarized when treated with chromanol 293B. V_m_ was detected using the fluorescence probe DisC3(5). The fluorescence intensity was directly proportional to V_m_. As shown in Figure 4B, V_m_ of the chromanol 293B-treated groups increased during human sperm capacitation. The depolarized sperm V_m_ values for the groups treated with 100 and 200μM chromanol 293B were much higher than those for the vehicle control group (*p*<0.05). These results suggest that chromanol 293B increases [K^+^]_i_ and depolarizes V_m_ in human sperm.

### Chromanol 293B Decreases Intracellular Ca^2+^ Concentration During Human Sperm Capacitation

To explore whether the KCNQ1 channel indirectly influences other intracellular ion homeostasis, the effect of chromanol 293B on intracellular Ca^2+^ concentration ([Ca^2+^]_i_) was detected using the Ca^2+^ fluorescence probe Fluo3-AM, fluorescence intensity of which was directly proportional to [Ca^2+^]_i_. When human sperm were treated with chromanol 293B, the [Ca^2+^]_i_ decreased compared to the vehicle control at the beginning of 25min (*p*<0.05; Figure 4C). The effect of 200μM chromanol 293B on [Ca^2+^]_i_ decrease was negligible compared to that of 100μM chromanol 293B. However, both 100 and 200μM chromanol 293B groups showed a significant decrease in [Ca^2+^]_i_ compared to the vehicle control group (*p*<0.05). These results indicate that KCNQ1 indirectly regulates [Ca^2+^]_i_ during human sperm capacitation. After capacitation for 3h, [Ca^2+^]_i_ increased significantly compared to the beginning of the experiment. However, there were no statistically significant differences between the groups at 3h. The explanation for this result may be that chromanol 293B initially inhibits KCNQ1, while other potassium channels compensate over time.

### Chromanol 293B Increases Intracellular Cl^−^ Concentration During Human Sperm Capacitation

The intracellular Cl^−^ concentration ([Cl^−^]_i_) was analyzed using the Cl^−^ fluorescence probe MQAE. The fluorescence intensity is inversely proportional to [Cl^−^]_i_. As shown in Figure 4D, within the first 25min, treatment with 200μM chromanol 293B significantly decreased the fluorescence intensity of Cl^−^, which indicating that [Cl^−^]_i_ increased (*p*<0.05; Figure 4D). Although there were no significant differences between all the groups when sperm were capacitated for 3h, the groups treated with 100 and 200μM chromanol 293B groups tended to have higher [Cl^−^]_i_. These results indicate that KCNQ1 indirectly regulates [Cl^−^]_i_ during human sperm capacitation.

### Chromanol 293B Decreases Intracellular pH During Human Sperm Capacitation

The intracellular pH (pH_i_) of human sperm during capacitation was measured using the pH fluorescence probe BCECF-AM. The fluorescence intensity was directly proportional to pH_i_. The chromanol 293B-treated groups showed a decrease in pH_i_ compared to that of the vehicle control group (Figure 4E).

## Discussion

Potassium channels play an essential role in sperm function. The membrane potential of sperm becomes hyperpolarized during capacitation, which is mainly due to the outflow of K^+^ currents (Lopez-Gonzalez et al., 2014). Patients lacking efflux K^+^ currents in sperm show reduced fertility (Brown et al., 2016). It is known that the K^+^ current characteristics in human sperm are different from those in mouse sperm. The K^+^ channel type in human sperm remains controversial (Mannowetz et al., 2013; Brenker et al., 2014). To identify the critical K^+^ channel types in sperm, gene knockout studies provide direct and credible evidence. However, this strategy cannot be used for studying human sperm. Other approaches, such as pharmacological assessment, immunolocalization, and patch clamp electrophysiology methods can be used to study K^+^ channels in human sperm (Mansell et al., 2014). Here, by using immunoblot, immunolocalization, and pharmacological methods, we first report that the KCNQ1 channel is expressed and localized in the head and tail regions of human sperm. Moreover, the KCNQ1 channel was observed to play a role in regulating human sperm motility, AR, protein tyrosine phosphorylation, and ion homeostasis during capacitation.

The human KCNQ1 pore-forming subunit contains 676 amino acids with a molecular weight of approximately 74.6kDa (Yang et al., 1997). Our Western blotting experiment using KCNQ1-specific antibodies showed a clear band at approximately 70kDa (Figure 1A), demonstrating that KCNQ1 is expressed in human sperm. Using RT-PCR, Yeung et al. found that one of the KCNQ1 auxiliary subunits, KCNE1, is expressed in human sperm. Immunofluorescence showed that this subunit localized in the tail regions and cytoplasmic droplets of human sperm (Yeung and Cooper, 2008). Human KCNE1 contains 129 amino acids with a molecular weight of approximately 14.6kDa (Tian et al., 2007). Evaluation of KCNE1 expression in human sperm by Western blotting revealed a specific band at approximately 15kDa (Figure 1A), indicating that KCNE1 was expressed in human sperm. We found that KCNQ1 is localized in the head, neck, and tail regions of human sperm, while KCNE1 is localized in the neck and tail regions, as previously reported (Yeung and Cooper, 2008; Figure 1B). KCNQ1 and KCNE1 showed partial co-localization in the human sperm. The distribution of the two proteins showed no obvious differences between the non-capacitation and capacitation groups. These results suggest that KCNQ1 may form a functional channel in human sperm.

Because KCNQ1 is expressed in human sperm, we further investigated its role in sperm capacitation using pharmacological assessments. Chromanol 293B is a specific inhibitor of KCNQ1 which can electrostatically interact with the selectivity filter of KCNQ1 and block the channel (Lerche et al., 2007). The half-maximal inhibitory concentration (IC50) of chromanol 293B on KCNQ1 is approximately 65μM. The KCNE family of auxiliary subunits enhances this block. The IC50 of chromanol 293B on KCNQ1/KCNE1 is approximately 15μM (Bett et al., 2006). Here, we treated human sperm with 20, 100, or 200μM chromanol 293B to examine the role of KCNQ1 in human sperm capacitation.

We analyzed the AR, hyperactivation, and protein tyrosine phosphorylation in human sperm treated with chromanol 293B because these phenomena occur during sperm capacitation. The results show that 100μM chromanol 293B significantly reduced the AR ratio of human sperm after capacitation for 3h (29.7±1.4% vs. 35.1±1.7%, *p*=0.017), with no influence on sperm viability (Figure 2). In addition to KCNQ1 potassium channels, there are other potassium channels in human sperm, such as Slo1 and Slo3. These may play a compensatory role when KCNQ1 potassium channels are inhibited. Sperm treated with 100μM chromanol 293B showed lower sperm motility and progressive motility. Treatment with 200μM chromanol 293B significantly reduced sperm motility parameters, including sperm motility, progressive motility, VAP, VSL, VCL, BCF, STR, and LIN. However, the hyperactivation of human sperm in the chromanol 293B-treated groups and vehicle control was not significantly different (Table 1). This may also be because Slo1 or Slo3 potassium channels in human sperm play compensatory roles when KCNQ1 is inhibited. Many researchers have found that protein tyrosine phosphorylation is associated with sperm capacitation (Varano et al., 2008; Dona et al., 2011; Vyklicka and Lishko, 2020). Our research showed that protein tyrosine phosphorylation levels in the vehicle control group increased significantly after capacitation for 3h. However, chromanol 293B decreased protein tyrosine phosphorylation levels in a dose-dependent manner after capacitation for 3h (Figure 3B), suggesting that KCNQ1 is involved in human sperm function regulation. Chromanol 293B also changed the localization of phosphorylated tyrosine, reducing its levels in the equatorial region of human sperm (Figure 3C). Recently, Dona et al. found that the Cl^−^/HCO_3_^−^ exchanger SLC4A1(AE1) can be directly phosphorylated by Src family kinases, and the SLC4A1-Tyr-phosphorylation level in the apical region of sperm is involved in sperm capacitation. The inhibitors of SLC4A1 reduced phosphorylated tyrosine levels in the head of human sperm, which is critical for AR (Dona et al., 2020). Other researchers have also found that protein tyrosine phosphorylation in the sperm head region is relevant to AR (Dona et al., 2011; Andrisani et al., 2015). Therefore, chromanol 293B changed the level and localization of protein tyrosine phosphorylation, which influenced the function of human sperm; however, the specific mechanisms remain unknown.

**Figure 5:**
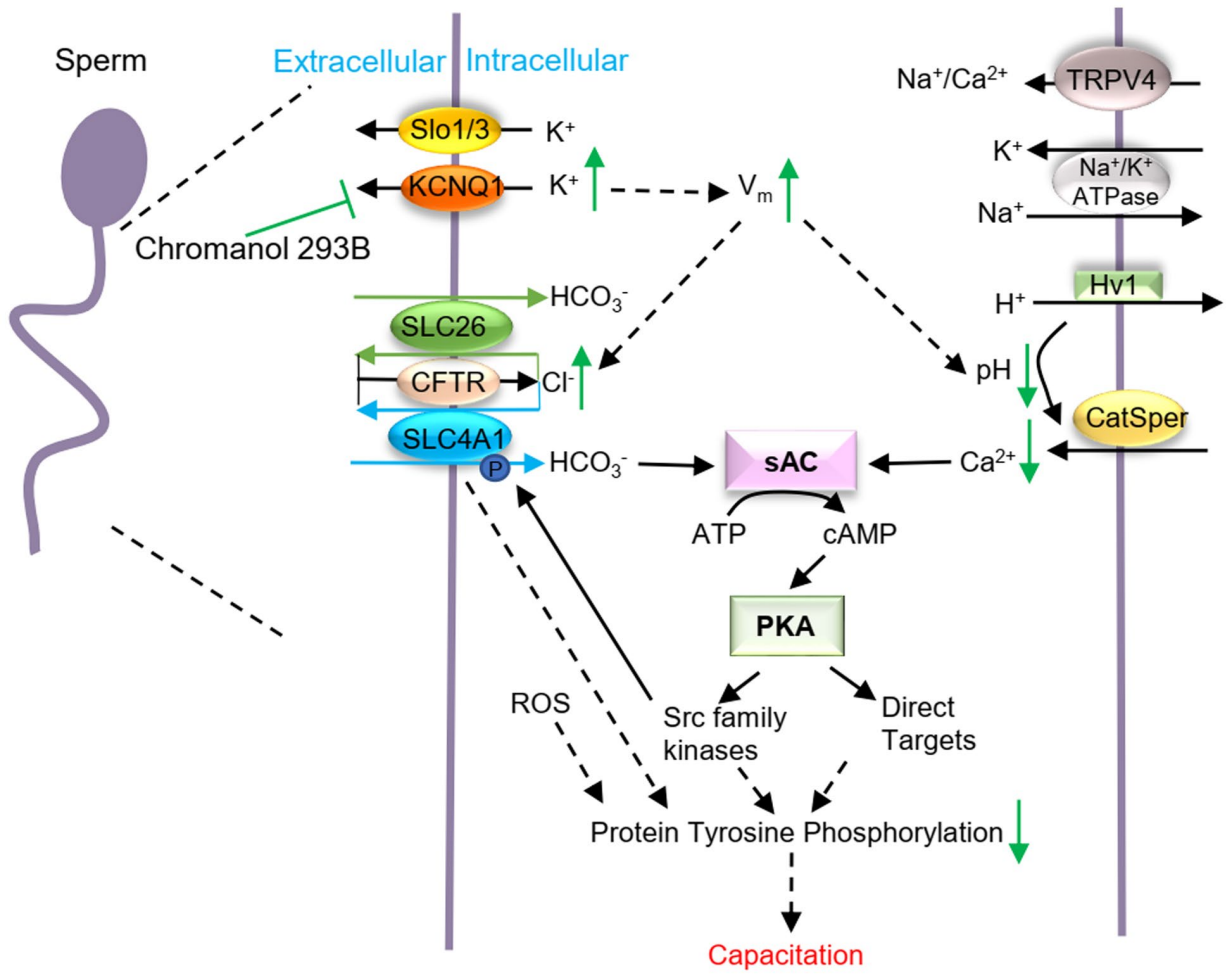
Schematic representation of how KCNQ1 potassium channel regulates human sperm function. When human sperm are treated with chromanol 293B, the K^+^ outflow currents mediated by KCNQ1 are inhibited, which causes a [K^+^]_i_ increase and the membrane depolarization. V_m_ changes regulate other voltage-gated channels and affect the electrical driving force for ions. Thus, [Cl^−^]_i_ increases, while the pH_i_ and [Ca^2+^]_i_ decreases. It is generally known that during capacitation, Ca^2+^ and HCO_3_^−^ activate soluble adenylate cyclase (sAC) which converts ATP into cAMP. Then PKA can be activated. PKA subsequently activates target proteins and protein tyrosine phosphorylation levels increase. KCNQ1 in human sperm participates in ion homeostasis regulation and affects capacitation.

K^+^ is involved in V_m_ regulation and its retention in sperm causes the [K^+^]_i_ to increase. During human sperm capacitation, V_m_ is always hyperpolarized, which is mainly due to the efflux of K^+^ (Lopez-Gonzalez et al., 2014). We found that chromanol 293B depolarized human sperm V_m_ (Figure 4B), demonstrating that it inhibits KCNQ1 and increases [K^+^]_i_ and V_m_ (Figure 5). V_m_ then regulates ion homeostasis, as some channels in sperm are voltage-gated, and ions are also regulated by potential and chemical driving forces.

Ca^2+^ is the most important ion in sperm function during capacitation. During human sperm capacitation, [Ca^2+^]_i_ increases (Correia et al., 2015). We found that chromanol 293B decreased the [Ca^2+^]_i_ (Figure 4C). This may be because KCNQ1 inhibition causes V_m_ to increase, which then reduces the electrical driving force for Ca^2+^ influx (Clapham, 2013). Notably, Ca^2+^ is critical for sperm motility (Nowicka-Bauer and Szymczak-Cendlak, 2021), because it can bind to the motor protein dynein ATPase and regulate flagellar curvature (Wang et al., 2020). Thus, KCNQ1 inhibition decreases [Ca^2+^]_i_ and reduces sperm motility parameters. It is generally known that [Ca^2+^]_i_ and pH_i_ increase during human sperm capacitation. Ca^2+^ and HCO_3_^−^ activate soluble adenylate cyclase (sAC), which converts ATP into cAMP. Protein kinase A (PKA) is subsequently activated and regulates its target proteins, and protein tyrosine phosphorylation levels increase (Allouche-Fitoussi and Breitbart, 2020; Figure 5). The decrease in [Ca^2+^]_i_ induced by chromanol 293B may explain the decrease in protein tyrosine phosphorylation levels during capacitation.

Furthermore, our results indicate that KCNQ1 inhibition increased [Cl^−^]_i_ (Figure 4D). This may be because chromanol 293B depolarizes sperm membrane and then increases the electrical driving force for Cl^−^. The increasing of [Cl^−^]_i_ in our study indicates that Cl^−^ influx is greater than Cl^−^ efflux. In human sperm, the anion channel cystic fibrosis transmembrane conductance regulator (CFTR) mediates the influx of Cl^−^ (Xu et al., 2007). Solute carrier 26 (SLC26) family and SLC4A1 in human sperm are Cl^−^/HCO_3_^−^ exchangers. Chan et al. proposed that SLC26 family members take up HCO_3_^−^ and export Cl^−^ with the interaction of CFTR. CFTR can provide the Cl^−^ through a recycling pathway (Chan et al., 2009; Chan and Sun, 2014; Puga Molina et al., 2018). Therefore, less Cl^−^ outflow accompanies less HCO_3_^−^ influx, which leads to decreasing of intracellular pH and also disturbs sperm capacitation through sAC/cAMP/PKA pathway (Figure 5). Bachmann et al. found that chromanol 293B also blocked CFTR, with the IC50 fivefold higher than inhibition of KCNQ1/KCNE1 (Bachmann et al., 2001). However, some researchers observed no effect of chromanol 293B on Cl^−^ currents mediated by CFTR (Alzamora et al., 2011; Shimizu et al., 2014). CFTR inhibition theoretically reduces Cl^−^ influx and decreases [Cl^−^]_i_; however, our experiment showed that chromanol 293B increases [Cl^−^]_i_. These results suggest that chromanol 293B inhibits the KCNQ1 channel and indirectly regulates [Cl^−^]_i_ in human sperm. Others have also found that KCNQ1 indirectly regulates Cl^−^ secretion *via* CFTR in colonic cells by changing the electrical driving force, which is in accordance with our research (Preston et al., 2010; Alzamora et al., 2011).

Cytoplasmic alkalinization is another characteristic of sperm capacitation (Matamoros-Volante and Treviño, 2020). When human sperm were treated with chromanol 293B, the pH_i_ of the sperm decreased (Figure 4E), and such a decrease is not conducive to capacitation. In human sperm, the most important Ca^2+^ channel, CatSper, is activated by progesterone, membrane potential depolarization and cytoplasmic alkalinization (Sun et al., 2017). Chromanol 293B- mediated reduction in pH_i_ may further inhibit Ca^2+^ influx through CatSper and cause a decrease in [Ca^2+^]_i_, as observed in the present study.

In summary, we found that KCNQ1 is expressed in human sperm, localized in the head and tail regions and is partially co-localized with KCNE1. KCNQ1 participates in the regulation of ion homeostasis and affects the AR, sperm motility, and protein tyrosine phosphorylation during human sperm capacitation. It is likely that when KCNQ1 is inhibited in human sperm, the K^+^ efflux is blocked, and the membrane potential is depolarized. The increase in membrane potential changes the electrical potential driving force of other ions and may influence other voltage-gated ion channels. Thus, ion homeostasis in human sperm is altered, including [Ca^2+^]_i_ and pH_i_ which are critical for capacitation (Figure 5). KCNQ1 is not the only potassium channel expressed in human sperm; there are many other important potassium channels, such as Slo1 and Slo3. These potassium channels may be able to help compensate if KCNQ1 is not fully functional. To verify the activity of the KCNQ1 channel in human sperm, its role in genetic male infertility should be studied in the future.

## Data Availability Statement

The raw data supporting the conclusions of this article will be made available by the authors, without undue reservation.

## Ethics Statement

The studies involving human participants were reviewed and approved by Ethics committee of Hangzhou Medical College. The patients/participants provided their written informed consent to participate in this study.

## Author Contributions

PS designed the study. TG, KL, FL, JY, AL, YN, and PS performed the experiments. PS and TG analyzed the data and drafted the paper. All authors read and approved the submitted and final versions.

## Funding

This research was funded by National Natural Science Foundation of China (Nos. 81801525 and 81771647), Health Sci&Tech Plan Project of Zhejiang Province (Nos. 2018KY039 and 2019KY363), Natural Science Foundation of Zhejiang Province (No. LQ17H040004), Special Project for the Research Institutions of Zhejiang Province (Nos. YS2021014, C11920D-04 and YS2021011), Zhejiang Province Program for the Cultivation of High-level innovative Health Talents (Year 2018).

## Conflict of Interest

The authors declare that the research was conducted in the absence of any commercial or financial relationships that could be construed as a potential conflict of interest.

## Publisher’s Note

All claims expressed in this article are solely those of the authors and do not necessarily represent those of their affiliated organizations, or those of the publisher, the editors and the reviewers. Any product that may be evaluated in this article, or claim that may be made by its manufacturer, is not guaranteed or endorsed by the publisher.
