# Supplementary material for: KCNQ1 Potassium Channel Expressed in Human Sperm Is Involved in Sperm Motility, Acrosome Reaction, Protein Tyrosine Phosphorylation, and Ion Homeostasis During Capacitation
Source: Front Physiol. 2021 Oct 22;12:761910. doi: 10.3389/fphys.2021.761910 (PMC8569670; doi:10.3389/fphys.2021.761910)
Supplement: Supplementary file 1 [file Data_Sheet_1.DOCX]

Supplementary Material

# Supplementary Figures and Tables

## Supplementary Table

# Table S1. Supplementary material of Figure 4. Exact P value between samples and vehicle control at the same time point. Only P<0.05 were shown.

|  |  | P value | P value | P value |
| --- | --- | --- | --- | --- |
|  | time（min） | 20 μM 293B | 100 μM 293B | 200 μM 293B |
| [K^+^]_i_ | 3 |  |  | 0.032 |
| [K^+^]_i_ | 18 |  |  | 0.021 |
| [K^+^]_i_ | 27 |  | 0.019 | 0.020 |
| [K^+^]_i_ | 30 |  | 0.020 | 0.032 |
| V_m_ | 9 |  | 0.003 | 0.004 |
| V_m_ | 12 |  | 0.005 | 0.018 |
| V_m_ | 15 |  | 0.008 | 0.031 |
| V_m_ | 18 |  | 0.028 |  |
| V_m_ | 21 |  | 0.003 | 0.007 |
| V_m_ | 24 |  | 0.017 | 0.027 |
| V_m_ | 27 |  | 0.017 | 0.028 |
| V_m_ | 30 |  | 0.043 | 0.028 |
| V_m_ | 180 |  | 0.000 | 0.000 |
| [Ca^2+^]_i_ | 3 | 0.011 | 0.000 | 0.004 |
| [Ca^2+^]_i_ | 6 |  | 0.037 | 0.037 |
| [Ca^2+^]_i_ | 9 |  | 0.025 | 0.013 |
| [Ca^2+^]_i_ | 12 |  | 0.014 | 0.011 |
| [Ca^2+^]_i_ | 15 | 0.026 | 0.007 | 0.008 |
| [Ca^2+^]_i_ | 18 | 0.030 | 0.002 | 0.001 |
| [Ca^2+^]_i_ | 21 |  | 0.008 | 0.006 |
| [Ca^2+^]_i_ | 24 |  | 0.006 | 0.002 |
| [Cl^-^]_i_ | 3 |  |  | 0.000 |
| [Cl^-^]_i_ | 6 |  |  | 0.001 |
| [Cl^-^]_i_ | 9 |  | 0.009 | 0.000 |
| [Cl^-^]_i_ | 12 |  | 0.031 | 0.000 |
| [Cl^-^]_i_ | 15 |  | 0.026 | 0.000 |
| [Cl^-^]_i_ | 18 |  | 0.026 | 0.000 |
| [Cl^-^]_i_ | 21 |  |  | 0.000 |
| [Cl^-^]_i_ | 24 | 0.030 | 0.013 | 0.000 |
| [Cl^-^]_i_ | 27 |  |  | 0.000 |
| pH_i_ | 18 |  |  | 0.042 |
| pH_i_ | 180 |  | 0.025 | 0.001 |

## Supplementary Figures

#
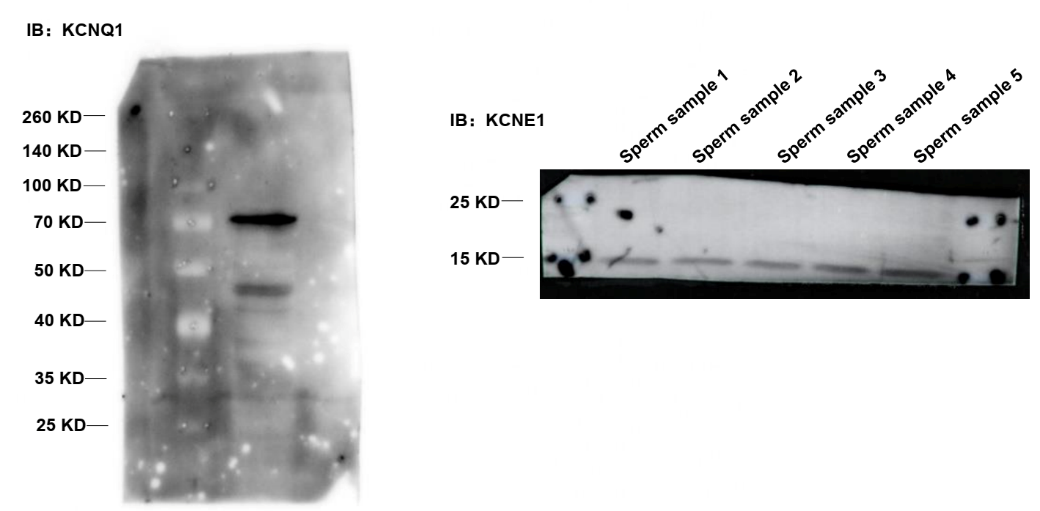


# Supplementary Figure 1. Full uncropped immunoblots of Figure 1. Human sperm were lysed after capacitation for 3 h. Sperm proteins were separated using SDS-PAGE and analyzed by western blotting with antibodies for KCNQ1 and KCNE1.

#



# Supplementary Figure 2. Full uncropped immunoblots of Figure 3. Western blotting results showing the effect of chromanol 293B on protein tyrosine phosphorylation during human sperm capacitation. Sperm before capacitation incubation were set as control. Other sperm were treated with the vehicle control (DMSO), 20 μM chromanol 293B and 100 μM chromanol 293B under capacitated conditions for 30 min and 3 h. Thereafter, sperm were lysed and the proteins were resolved by SDS-PAGE. The protein tyrosine phosphorylation was detected using a primary anti-phosphotyrosine antibody by western blotting. Subsequently, the blot was stripped and probed with an anti-β-tubulin antibody as a loading control.
